# Reply to Monteil et al.: Universal presence of core magnetite biomineralization genes points to ancient symbiosis

**DOI:** 10.1073/pnas.2210188119

**Published:** 2022-08-29

**Authors:** M. Renee Bellinger, Uwe Hartmann, Hervé Cadiou, Michael Winklhofer, Michael A. Banks

**Affiliations:** ^a^Department of Biology, University of Hawai‘i at Hilo, Hilo, HI 96720;; ^b^Experimental Physics Department, Saarland University, D-66041 Saarbruecken, Germany;; ^c^Institut des Neurosciences Cellulaires et Intégratives, CNRS UPR3212, F-67100 Strasbourg, France;; ^d^Institute of Biology and Environmental Science, University of Oldenburg, D-26129 Oldenburg, Germany;; ^e^Research Center Neurosensory Science, University of Oldenburg, D-26111 Oldenburg, Germany;; ^f^Coastal Oregon Marine Experiment Station, Dept. Fisheries & Wildlife, Hatfield Marine Science Center, Oregon State University, Newport, OR 97365

Monteil et al. (1) comment that, although the hypothesis of ancient symbiotic events leading to transfer of magnetite biomineralization genes (MBGs) from magnetotactic bacteria (MTB) to eukaryotes has been raised for decades (2), Bellinger et al. (3) do not provide evidence supporting that MBGs are, per se, functionally equivalent to magnetosome genes homologs. Obvious hypothesis precursors include quantifying whether eukaryote genomes contain the necessary genetic machinery, that is, suites of distant homologs of MTB magnetosome biomineralization genes, and associating those with magnetite production. Accordingly, we (3) examined genomes of 13 phylogenetically diverse eukaryotes, many known for keen navigational sense, and the Asgard archea clade Lokiarchaeota. If MBGs were absent in some or all, then production of biogenic magnetite across diverse life forms could only be explained by convergent evolution. However, our findings point to a different path in support of ancient symbiotic events: Distant homologs of MBGs are universally present in eukaryote genomes (and Lokiarchaeota), including four of five core genes universally shared by MTB Nitrospirae and Proteobacteria (4), composing a subset of the minimal set of genes required for magnetosome biomineralization in prokaryotes (5, 6). Our genetic hypothesis testing was then extended to include transcriptomics data from candidate magnetite-based magnetoreceptors contained in salmonid olfactory tissues (7). That distant homologs of MBGs were differentially and more highly expressed in magnetic relative to nonmagnetic olfactory cells is compelling evidence for our bold, yet not unprecedented (2), suggestion: These genes are components of a common, ancient genetic mechanism utilized for magnetite production. It is unsurprising that our needle in a haystack search revealed “few magnetosome genes,” as asserted by ref. 1, considering those specific gene targets comprised ≪1% of all possible salmonid proteins, and MTB versus salmonid genome complexity. For example, genome sizes of MTB *Magnetospirillum gryphiswaldense* (8) and Chinook salmon (9) are ∼4.16 megabases versus ∼2.29 gigabases, with corresponding numbers of total proteins, 3,903 versus 49,936. Thus, our interpretation is hardly “a biased overinterpretation” (1).

Likewise, the observation that “homologs like the selected 11 overexpressed genes were also underexpressed” (1) is unsurprising. Whole genome duplication events produced eight copies of the original deuterostome genome in ray-finned fish (10), salmonids underwent an additional genome duplication, and orthologs and paralogs functionally diverge over time (9). Further, the statement that “mandatory genes/motifs for the magnetite biomineralization like the unique magnetochrome motif are absent in the salmon transcriptome” (1) is not strictly correct. Although transcripts containing the cytochrome motif CXXCH (11) were absent from the differentially expressed gene dataset, that motif is common among salmonid proteins, occurring ∼144 times within the Chinook salmon genome (3). Functional compensation through interactions with other (nondifferentially expressed) genes is certainly possible.

We agree with ref. 1 that ancient symbiosis leading to transfer of magnetite biomineralization genetic machinery from MTB to eukaryotes has long been raised as a good idea, and further their assertion that experimental evidence to determine whether functional counterparts involved in biomineralization are involved in magnetic sensory transduction is critical to resolving the genetic underpinnings of this fascinating sensory process.
